# Molecular mechanisms underlying the role of hypoxia-inducible factor-1 α in metabolic reprogramming in renal fibrosis

**DOI:** 10.3389/fendo.2022.927329

**Published:** 2022-07-25

**Authors:** Xuejiao Wei, Yue Hou, Mengtuan Long, Lili Jiang, Yujun Du

**Affiliations:** ^1^ Department of Nephrology, The First Hospital of Jilin University, Changchun, China; ^2^ Department of Physical Examination Center, The First Hospital of Jilin University, Changchun, China

**Keywords:** hypoxia-inducible factor-1α, aerobic glycolysis, oxidative phosphorylation, metabolic reprogramming, renal fibrosis

## Abstract

Renal fibrosis is the result of renal tissue damage and repair response disorders. If fibrosis is not effectively blocked, it causes loss of renal function, leading to chronic renal failure. Metabolic reprogramming, which promotes cell proliferation by regulating cellular energy metabolism, is considered a unique tumor cell marker. The transition from oxidative phosphorylation to aerobic glycolysis is a major feature of renal fibrosis. Hypoxia-inducible factor-1 α (HIF-1α), a vital transcription factor, senses oxygen status, induces adaptive changes in cell metabolism, and plays an important role in renal fibrosis and glucose metabolism. This review focuses on the regulation of proteins related to aerobic glycolysis by HIF-1α and attempts to elucidate the possible regulatory mechanism underlying the effects of HIF-1α on glucose metabolism during renal fibrosis, aiming to provide new ideas for targeted metabolic pathway intervention in renal fibrosis.

## Introduction

Chronic kidney disease (CKD) is a global public health concern. With an increase in hypertension and diabetes, the prevalence of CKD is increasing every year (1). Renal fibrosis is a common pathological change in the transition from CKD to end-stage renal disease caused by different pathological changes, mainly manifesting as glomerulosclerosis, renal tubular atrophy, and renal interstitial fibrosis, which are closely related to renal deterioration (2). Currently, there is no effective treatment for renal fibrosis; thus, it is of great clinical significance to find a way to improve or reverse renal fibrosis. Abnormal energy metabolism, often considered a marker of cancer cells, has attracted considerable attention in this research area. In contrast to normal cells, which derive most of the energy from mitochondrial oxidative phosphorylation (OXPHOS), cancer cells rely on aerobic glycolysis as the main energy source (3). This metabolic pattern, similar to that in tumor cells, has also been confirmed in damaged kidneys, and aerobic glycolysis may be a possible mechanism driving renal fibrosis (4). The fibrotic kidney undergoes metabolic reprogramming to adapt, and activating aerobic glycolysis is a way for kidney cells to cope with metabolic stress (5).

Hypoxia-inducible factor-1 α (HIF-1α) is the main regulator of cell responses under hypoxia. The α subunit of HIF-1 binds to the β subunit to form a dimer, which then binds to hypoxia response elements (HRE) in promoters or enhancer regions of downstream target genes to initiate gene transcription (6). Transforming growth factor-β1 (TGF-β1) is the main cytokine that promotes renal fibrosis, and inhibition of TGF-β1 or its downstream signaling pathway can significantly inhibit renal fibrosis (7). The HIF-1α transcription complex induces TGF-β1 activation (8). Subsequently, TGF-β1 stabilizes HIF-1α by selectively inhibiting proline hydroxylase-2 (9). A mutual regulatory relationship between HIF-1α and TGF-β1 promotes renal fibrosis. Furthermore, studies have shown that HIF-1α is an important transcription factor that inhibits mitochondrial OXPHOS and activates the glycolysis pathway, regulating aerobic glycolysis by transcriptional regulation of a series of glycolytic genes (10).

In this review, we summarized the latest knowledge on the mechanisms of action and function of HIF-1α in regulating glucose metabolism reprogramming during renal fibrosis, focusing on the regulation of glycolysis-related enzymes. We hope to provide a new strategy for targeted therapy of renal fibrosis.

## Metabolic reprogramming in renal fibrosis

Renal fibrosis is a common result of various CKDs, regardless of the underlying cause. Currently, it is believed that renal fibrosis is not merely a static and substantial scar, but a dynamic and evolving process involving multiple complex signaling pathways and intercellular crosstalk. The main manifestations are the release of inflammatory cytokines, accumulation of extracellular matrix (ECM), transformation of renal intrinsic cells to mesenchymal cells, activation of fibrosis-related signaling pathways, and renal microvascular lesions (11, 12). Although a multitude of experimental data are available regarding targeting the above pathogenic processes, the application of anti-fibrotic therapeutic strategies to patient treatment still needs further exploration. Finding more effective and targeted methods to improve renal fibrosis is of great significance.

The change in energy metabolism is increasingly regarded as an important pathogenic mechanism in CKD, and normal energy metabolism is the basis for maintaining the physiology of the kidney (13). Although the kidney is not a metabolic organ, it requires a large amount of energy to filter waste, regulate electrolyte and fluid balance, and perform other processes (14). On the one hand, abnormal energy metabolism leads to renal fibrosis. On the other hand, kidney damage alters energy metabolism. Thus, abnormal energy metabolism and renal fibrosis cause and affect each other, forming a vicious cycle (13).

Most cells rely on mitochondrial OXPHOS for energy production, whereas glycolysis occurs only when oxygen is scarce. Glycolysis is the production of adenosine triphosphate (ATP) through a series of intercellular enzymatic reactions that require ten enzymes, including three rate-limiting enzymes, hexokinases (HKs), phosphofructose kinase 1 (PFK1), and pyruvate kinase M2(PKM2), which catalyze irreversible reactions (15). Under the action of ten enzymes, glucose is gradually degraded to pyruvate, resulting in the formation of two ATP molecules. Pyruvate is reduced to lactate by lactate dehydrogenase-A(LDHA) when the oxygen supply is insufficient. In the presence of oxygen, pyruvate is converted to acetyl-CoA, which then enters the tricarboxylic acid cycle (TCA cycle) to produce nicotinamide adenine dinucleotide (NADH) and flavin adenine dinucleotide (FADH_2_), which enters the electron transport chain (ETC) to promote OXPHOS to produce ATP (15). However, this ubiquitous pathway does not seem to be applicable to tumor cells. Warburg found that even under normoxia, cancer cells could not use mitochondrial OXPHOS to obtain energy, and that pyruvate could not enter the TCA cycle but was instead degraded by LDHA into lactate for energy. This discovery changed our previous understanding of cancer cell metabolism, and the phenomenon was later termed aerobic glycolysis or the “Warburg effect” (16). Subsequently, the Warburg effect was observed and confirmed in various tumor cells. Moreover, continuous studies have shown that the Warburg effect, which may be the mechanism driving renal fibrosis, is also observed in injured kidneys (17). Blocking aerobic glycolysis inhibits renal fibrosis progression. Targeted metabolic analysis of renal tissues of rats with unilateral ureteral obstruction (UUO) showed high levels of lactate in renal tissue (18). This suggests that glycolysis is enhanced in the fibrotic kidney.

Under physiological conditions, proximal renal tubular epithelial cells (TECs) account for 90% of the renal cortex, which is rich in mitochondria, mainly uses fatty acids as fuel, and produces ATP through mitochondrial OXPHOS. When the kidney is damaged, TECs alter their energy metabolism and rely on aerobic glycolysis to adapt to the destruction of mitochondrial OXPHOS. This abnormal change of energy metabolism promotes renal fibrosis (19). Abnormal proliferation of TECs in the UUO model, which is characterized by high levels of glycolytic enzymes, was consistent with the degree of fibrosis (20). Cao et al. found that the activation of mammalian target of rapamycin (mTOR) signaling mediated by tuberous sclerosis complex 1 (a key regulator of glycolysis) promoted TEC proliferation and glycolytic enzyme upregulation, as well as extensive renal interstitial fibrosis, which was significantly improved using a glycolysis inhibitor (21). Zhang et al. observed that in UUO mice and TECs in CKD patients, OXPHOS was interrupted, glycolysis was increased, and mitochondrial proteins were highly acetylated, whereas deacetylation of pyruvate dehydrogenase complex (PDC), a key enzyme linking glycolysis to the TCA cycle, at lysine 385 could be significantly reversed, thereby improving renal fibrosis (22).

Studies have confirmed that the activation of renal interstitial fibroblasts is similar to metabolic reprogramming in cancer cells, and enhanced aerobic glycolysis motivates fibroblast-myofibroblast transformation, leading to renal fibrosis, which is reduced after the use of glycolysis inhibitors (23). In UUO kidneys or TGF-β1-induced renal interstitial fibroblasts, the expression of glycolysis-related enzymes was upregulated and aerobic glycolytic flux (glucose uptake and lactate production) was increased, which was positively correlated with the degree of fibrosis (20). Wei et al. observed that glycolytic genes were upregulated in renal interstitial fibroblasts in UUO mice, and when treated with glycolysis inhibitors, the production of fibronectin and α-smooth muscle actin was reduced, and the degree of renal fibrosis was alleviated (24).

## HIF-1α regulatory mechanism and biological effects

Hypoxia-inducible factors (HIFs) are important heterodimer transcription factors that sense oxygen status. They have an oxygen-sensitive α subunit, which plays the main biological role, and a stably expressed β subunit (25). HIFs perform their biological functions as follows. When oxygen is sufficient, the prolyl residue on the α subunit is hydroxylated by prolyl hydroxylase domain (PHD), combines with the VHL protein, recruits ubiquitin ligases, enters the proteasome for degradation, preventing HIF from exerting its biological function (26). Under hypoxia, hydroxylation of the α subunit is inhibited, and HIF-α instead of being degraded it accumulates. Subsequently, it enters the nucleus, combines with the β subunit to form a dimer, which binds to the HRE of the downstream target gene promoter or enhancer region, and activates the transcription of all target genes (26). Excluding the PHD, the stability and transcriptional activities of HIF-α are regulated by a factor inhibiting hypoxia-inducible factor. The factor inhibiting hypoxia-inducible factor is a JmjC domain 2-oxogluarate and Fe(II)-dependent oxygenase that catalyzes hydroxylation of specific asparagines in the C-terminal transcriptional activation domain of HIF-α isoforms. This modification suppresses the transcriptional activity of HIF by reducing its interaction with the transcriptional coactivators p300/CBP.

There are three α-subunit subtypes: HIF-1α, HIF-2α, and HIF-3α. Presently, there are many studies on HIF-1α and HIF-2α, the biochemical properties of which are very similar. They can identify the same DNA binding region and play a key role in the regulation of the cell hypoxia response (25). However, reports on HIF-3α are limited. Studies have shown that HIF-3α can be a HIF-1α target gene and negatively regulate the activities of HIF-1α and HIF-2α (27). In the kidney, HIF-1α exists in almost all cells, especially TECs, whereas HIF-2α is only expressed in specific cell types, such as vascular endothelial cells and renal interstitial fibroblasts (28).

HIF-1α has many important target genes that affect various physiological and pathological processes. Generally, the activation of HIF-1α manifests as two types of cellular adaptive responses. First, HIF-1α can promote angiogenesis by activating vascular endothelial growth factor (VEGF), Angiopoietin 2, notch ligand and ECM remodeling proteins, increasing the supply of oxygen and nutrients, and increasing the utilization rate of oxygen during metabolism (29). Moreover, it is worth noting that the activation of HIF-1α can also cause the upregulation of glucose transporter-1 (GLUT1) and glycolysis-related enzymes, which ultimately leads to the enhancement of aerobic glycolysis ability and inhibition of OXPHOS (10). Pyruvate produced through glycolysis is further metabolized to lactate by LDHA and excessive accumulation of lactate leads to intracellular acidosis. Monocarboxylate transporter 4, whose expression is HIF-1 dependent, plays a role in intracellular pH homeostasis during lactate efflux (30). Further, the expression and functional activation of carbonic anhydrase IX (CAIX) will increase as a coping mechanism (31). CAIX, a transmembrane protein neutralizing intracellular acidosis, is induced by HIF-1 and associated with glycolysis (32). Previous studies have reported that the expression of HIF-1α and its transcription target CAIX increases in tumor cells under hypoxia. This catalyzes the reversible conversion of carbon dioxide to bicarbonate and hydrogen ions and regulates the PH of tumors, leading to acidification of the tumor environment and alkalization of cytoplasm (33).

HIF-1 promotes glucose catabolism and glycogen synthesis. Pouysségur et al. determined that HIF-1 specifically upregulates enzymes involved in glycogen synthesis, such as phosphoglucomutase 1 and glycogen synthase 1, to maintain sufficient energy levels for later use under more severe nutrient-limiting conditions (34). Recently, Ito et al. demonstrated that HIF-1 protects the kidney from ischemia and hypoxia by enhancing glycogen synthesis and inhibiting glycogen decomposition in TECs (35). Fructose-1, 6-bisphosphatase 1 is among the major genes controlling renal gluconeogenesis. Li et al. demonstrated its ability to inhibit cell proliferation, glycolysis, and pentose phosphate pathways through direct interaction with the HIF “inhibitory domain” in pVHL (protein encoded by VHL gene) deficient renal cancer cells, thereby inhibiting nuclear HIF function and the potential Warburg effect.

Recent evidence indicates that many aspects of lipid metabolism are modified in a HIF-dependent manner during hypoxia. Peroxisome proliferator-activated receptor-γ, a member of the nuclear receptor transcription factor family, plays an important role in lipid metabolism by promoting free fatty acid uptake and triglyceride (TG) accumulation. Krishnan et al. demonstrated that HIF-1 can directly activate peroxisome proliferator-activated receptor-γtranscription, leading to the accumulation of TG in cardiomyocytes and promoting cardiomyocyte apoptosis (36). As a lipid metabolism disorder under hypoxia, the excessive accumulation of intracellular free fatty acids could cause lipotoxicity. Fatty acids can be converted to TG that is stored in the form of lipid droplets (LDs) in order to avoid this. TG synthesis is important for metabolic homeostasis and cell growth because LDs act as lipid stores providing both metabolic substrates and components for membrane synthesis. Triantafyllou et al. found that the expression of Acylglycerol-3-phosphate acyltransferase, an enzyme involved in the TG biosynthesis pathway, was directly regulated by HIF-1 and promoted the survival of cancer cells under hypoxia (37). Gimm et al. confirmed that HIF-1 can directly and specifically transactivate hypoxia-inducible protein 2, a novel LDs protein, to increase neutral lipid accumulation and stimulate cytokine expression in cancer cells (38).

## The regulatory mechanism of HIF-1α in renal fibrosis

**Figure 1:**
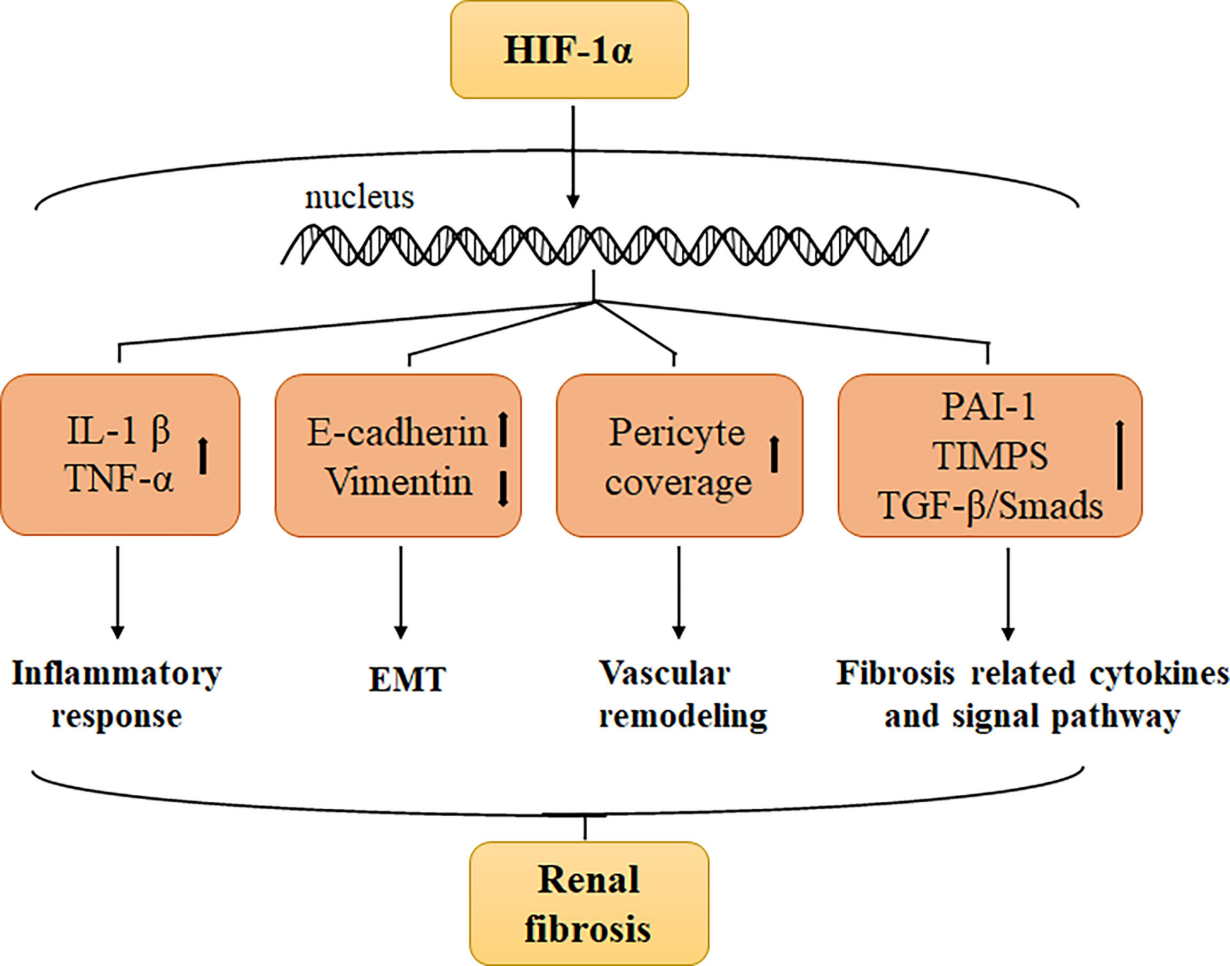
HIF-1α expression increases under hypoxia, which promoted renal fibrosis through the following aspects: Stimulating the secretion of interleukin-1β (IL-1 β) and tumor necrosis factor-α (TNF-α), and increasing the inflammatory reaction, upregulation of the epithelial cell marker E-cadherin and downregulation of the mesenchymal marker vimentin promote EMT, enhance pro-fibrosis cytokines such as plasminogen activator inhibitor 1 (PAI-1) and tissue inhibitor of metalloproteinase (TIMPs), and regulate fibrosis-related signaling pathways such as TGF-β/Smads signaling.

A series of studies have confirmed that the expression of HIF-1α increases in renal fibrosis, suggesting that HIF-1α may play a vital role in promoting renal fibrosis (39). How does HIF-1α mediate renal fibrosis? Presently, research has mainly focused on the role of HIF-1α in regulating the release of inflammatory mediators, promoting the epithelial-mesenchymal transition (EMT) process and renal vascular remodeling, activating the transcription of downstream pro-fibrosis-related cytokines, and interacting with various pro-fibrosis signaling pathways (40) (**Figure 1**). Notably, HIF-2 also plays a key role in the progress of renal fibrosis. Li et al. bred HIF-2α deficient mice and subjected them to renal trauma through UUO surgery, ultimately determining that HIF-2α ablation attenuated renal fibrogenesis induced by UUO injury (41). Studies on HIF-2 inhibiting renal fibrosis are ongoing, and long-term clinical trial data are expected to confirm the renoprotection and anti-fibrosis of HIF-2 in the future.

Inflammation exists widely in all stages of CKD and is an important factor in disease progression. Studies have shown that HIF-1α can release inflammatory mediators, such as interleukin-1β (IL-1β) and tumor necrosis factor-α (TNF-α), change the local microenvironment of the kidney, and cause inflammation and fibrosis (42). Studies have shown that TNF-α-induced nuclear factor-κB signaling stimulates muscle glycolytic metabolism through activation of the glycolytic regulator HIF-1α (43). Furthermore, inflammatory factors can upregulate HIF-1α in a nuclear factor-κB-dependent manner, thereby increasing the risk of kidney injury (44). Reactive oxygen species (ROS) are mainly produced by mitochondria, and they can mediate cell differentiation, proliferation, apoptosis and inflammatory responses by activating some signaling pathways (45). Inflammatory mediators such as TNF-α have been shown to induce ROS production (46). Recent studies have demonstrated that HIF-1α stabilization is regulated by ROS (47). Xie et al. investigated the relationship between TNF-α, ROS production and HIF-1α stabilization in hepatocellular carcinoma cells. Their results showed that TNF-α induced HIF-1α activation was ROS dependent, and inhibition of ROS generation significantly reduced HIF-1 α expression (48). Huang et al. found that titanium dioxide nanoparticle exposure increased ROS production and the expression of HIF-1α and TGF-β in TECs, which were reduced by ROS scavenger N-acetylcysteine treatment at the transcription level. These results suggested that titanium dioxide nanoparticle exposure may induce renal fibrosis through an ROS-related HIF-1α up-regulated TGF-β signaling pathway (49). Therefore, HIF-1α is closely related to inflammatory reactions that can promote the expression of inflammatory factors. Inflammatory reactions can also regulate the activity of HIF-1α.

EMT is an important process and a central link in the occurrence and development of renal fibrosis, in which enhancement of cell migration ability is an important feature of epithelial cell dedifferentiation (50). HIF-1α can activate the transcriptional activity of these genes by combining with HRE in the promoters of EMT regulators, such as Twist and Bmi1, downregulating the expression of E-cadherin and tight junction proteins, increasing the levels of fibronectin and vimentin, transforming epithelial cells into fibroblast-like cells, promoting EMT, and aggravating renal interstitial fibrosis (51, 52). Higgins et al. analyzed the regulatory effect of HIF-1α on the dedifferentiation of mouse TECs and proved that HIF-1α could enhance the migration ability of TECs, thus strengthening EMT. By selectively knocking out HIF-1α, collagen deposition, inflammatory infiltration, and fibroblast expression in the renal interstitium decreased (40).

A stable vascular network is important for maintaining tissue and organ homeostasis. Vascular remodeling is the adaptive response to various physiological and pathophysiological alterations that are closely related to aging and vascular diseases. The remodeling of the vascular network may initially be adaptive, however, eventually it becomes maladaptive and compromises organ function (53). Emerging evidence is suggesting that HIF-1α plays a pivotal role in vascular remodeling. Liu et al. found that interleukin-33 initiates vascular remodeling of hypoxic pulmonary hypertension by up-regulating HIF-1α and VEGF expression in vascular endothelial cells (54). Luo et al. showed that CD146 was up-regulated in pulmonary artery smooth muscle cells in proportion to disease severity, and CD146 expression and HIF-1α transcriptional program reinforced each other to drive vascular remodeling and pulmonary arterial hypertension (55). Accumulating evidence is demonstrating that the HIF-α hydroxylase system plays a critical role in vascular remodeling. Three PHD isoforms have been identified in mammals, PHD1, PHD2 and PHD3. Based on the expression pattern and dominant effects under normoxic conditions, PHD2 was considered as the critical oxygen sensor to hydroxylate HIF-α (56). Wang et al. confirmed that the deletion of endothelial PHD2 promoted renal vascular remodeling and fibrosis by up-regulating Notch3/TGF-β1 signaling and increasing the coverage of pericytes (57).Studies have shown that HIF-1α can combine with HRE in the promoters of plasminogen activator inhibitor 1 (PAI-1) and tissue inhibitor of metalloproteinase (TIMPs), trans-activate the transcription of target genes, and inhibit ECM degradation and collagen decomposition, thus promoting renal fibrosis (58, 59). TGF-β1 has long been regarded as a key molecule in the promotion of renal fibrosis, and the TGF-β/Smads signaling pathway is a classic profibrogenic signaling pathway. TGF-β1 mainly induces renal scar formation by activating downstream Smad signaling (7). Kushida et al. found that the increase in Smad3 binding was mainly caused by HIF-1α under hypoxia. Specifically, HIF-1α could bind to Smad3, upregulate the level of fibrosis-related proteins, and promote renal fibrosis (60). Arginase-II(Arg-II) is a mitochondrial enzyme which is highly expressed in renal proximal TECs and up-regulated by hypoxia. Recent studies have indicated that inhibition of Arg-II can reduce renal injury in diabetic nephropathy mouse models (61). Liang et al. (62) demonstrated that HIFs can mediate the up-regulation of Arg-II induced by hypoxia in TECs, and showed hypoxia promoting the expression of TGF-β1 dependent on Arg-II as the silence of Arg-II can prevent the expression of TGF-β1. These results indicate that hypoxia activates renal epithelial HIFs-Arg-II-TGF-β1 signaling, exacerbating renal injury and fibrosis.HIF-1α Promotes Glycolysis

**Figure 2:**
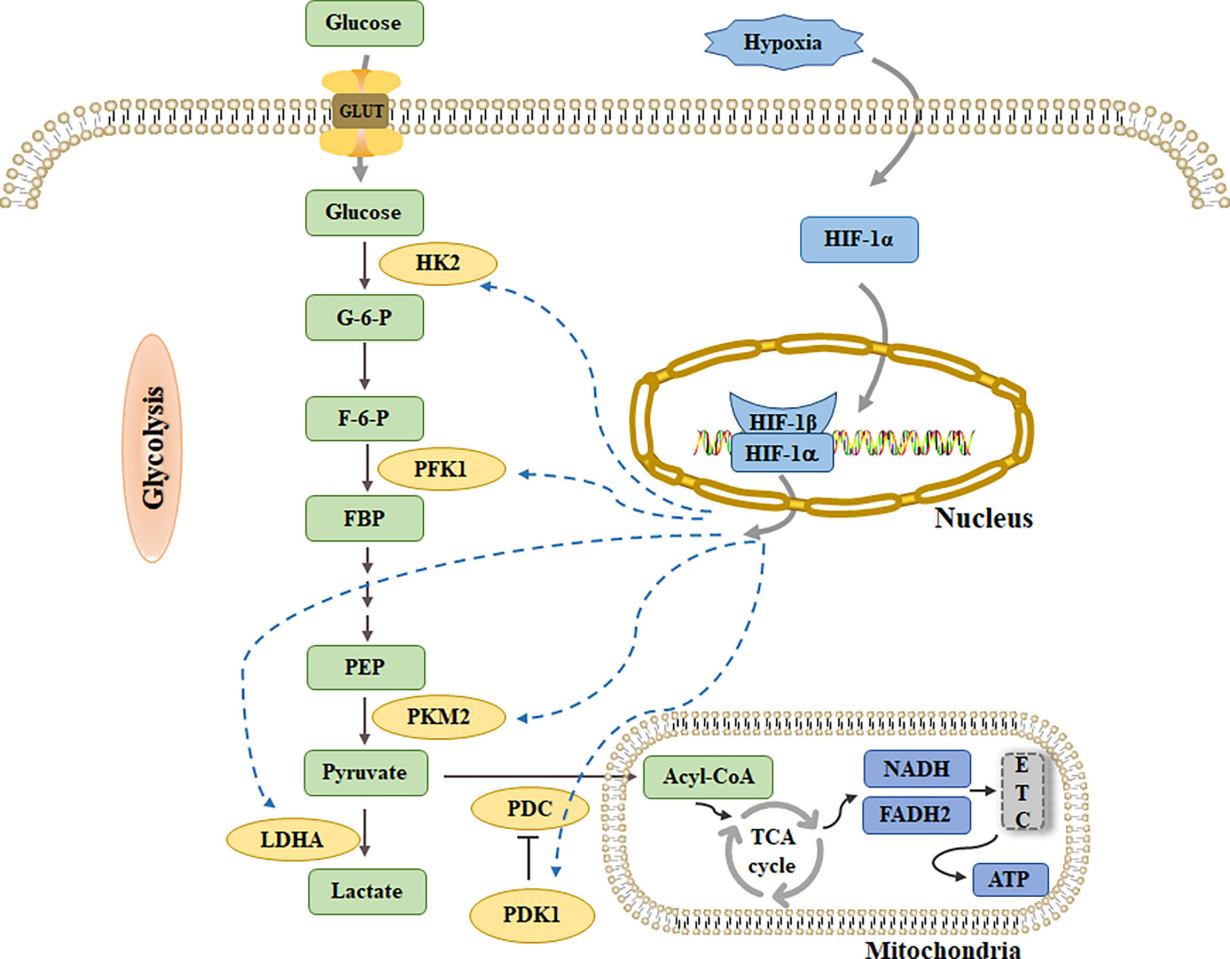
Glucose transport into cells through GLUT and decompose into pyruvate by a series of glycolytic enzymes such as HK2, PFK1 and PKM2. PDC catalyzes the conversion of pyruvate into acetyl-CoA, enters TCA cycle, generates NADH and FADH2, and then enters ETC to produce ATP. Under hypoxia, pyruvate is activated by LDHA to generate lactate. HIF-1α can increase glycolysis flux by up-regulating HK2, PFK1 and PKM2. At the same time, increasing the levels of PDK1 and LDHA inhibited pyruvate from entering TCA cycle.

One of the adaptive responses of cell survival mediated by HIF-1α is HIF-1α dependent glucose metabolism reprogramming. This metabolic reprogramming promotes an increase in glycolysis flux and inhibits OXPHOS by upregulating the expression of glucose transporters and glycolytic enzymes (10) (**Figure 2**). Studies have shown that genes encoding glycolytic enzymes, such as HKs, PFK1, aldolase, phosphoglyceride kinase-1, and LDHA, contain HIF-1α-binding sites in their promoter or enhancer regions, and are directly upregulated by HIF-1α in response to hypoxia (10). Interestingly, Semenza et al. observed that the consensus sequence recognized by HIF-1α in human aldolase and phosphoglyceride kinase-1 was evolutionarily conserved between humans and mice, which undoubtedly suggests that the HIF-1α-induced upregulation of these two genes would be of great significance in the regulation of glycolysis in mammals, and it warrants further study in the future (63).

### HIF-1α regulates GLUT1

HIF-1α precisely regulates the reabsorption and release of glucose into the blood by the kidneys. As glucose is soluble in water and cannot pass through the phospholipid bilayer of the cell membrane, carriers are required for its transport. Glucose transporters are cell membrane (transmembrane) glycoproteins that mediate extracellular glucose cell uptake. Three families of glucose transporters have been identified in humans: diffusion-prone glucose transporters (GLUTs) encoded by SLC2A, sodium-dependent glucose transporter encoded by SLC5A, and glucose efflux transporters encoded by SLC50A (64). In the kidney, only GLUTs and sodium-dependent glucose transporter are involved in the transport of renal glucose (64). To date, 14 subtypes of GLUTs (GLUT1–GLUT14) have been identified. Their distribution, function, and affinity with glucose differ, and they have different glucose transport efficiencies. GLUT1 and GLUT3 have the highest affinities for glucose, GLUT2 has the lowest affinity, and the glucose affinities of the rest are between those of GLUT1, 2, and 3. GLUT1 plays an important role in kidney and tumor cell metabolism and is closely related to glycolysis activity (65). In laryngeal carcinoma cells, the expression of GLUT1 is positively correlated with that of E-cadherin and negatively correlated with that of vimentin, indicating that GLUT1 is involved in EMT (66). Moreover, some studies have shown a correlation between GLUT1 expression and pathological fibrosis. Transgenic mice overexpressing GLUT1 showed an increase in the expression of TGF-β1 and nuclear factor-κB in the glomeruli and pathological manifestations similar to those in diabetic glomerulosclerosis (67). This finding suggests that GLUT1 may cooperate with cytokines or growth factors to induce renal fibrosis. The specific pathogenic mechanism involved requires further clarification.

GLUTs are one of the target genes activated by HIF-1α, but glucose transport into cells is undoubtedly detrimental to glycolysis (68). Studies have shown that an important way by which HIF-1α affects glycolysis is the upregulation of GLUTs. HIF-1α can induce the transcription and translation of SLC2A1 and SLC2A3, leading to the upregulation of GLUT1 and GLUT3, and finally to an increase in glycolysis flux. Wound healing and tumorigenesis are both involved in cell proliferation and tissue remodeling. Elson et al. found that HIF-1α and GLUT1 increased to their highest levels in re-epithelialized basal keratinocytes during the healing period of skin wounds. They believed that the increased GLUT1 level was related to the increased glucose uptake of cells, which was necessary to provide the energy needed for cell migration and proliferation during hypoxia (69).

GLUTs can effectively transport glucose into cells, provide substrates for glycolysis, and lay a foundation for glycolysis. Therefore, GLUTs will become a potential therapeutic target to limit the glycolysis rate and block energy sources. Presently, many GLUT1 inhibitors are in the research stage and potentially available, such as phloretin (the first discovered flavonoid GLUT1 inhibitor) (70) and resveratrol (71), which show good biological activities. Unfortunately, there is a lack of in-depth research on how HIF-1α regulates GLUT1 to affect energy metabolism in renal fibrosis. However, targeting GLUT1 also provides a new technical means for exploring the changes in energy metabolism in renal fibrosis.

### HIF-1α Regulates HK2

The first rate-limiting reaction in glycolysis is the phosphorylation of glucose to form glucose-6-phosphate (G-6-P), which is catalyzed by HKs. Four HKs (HK1, HK2, HK3, and HK4) have been identified in mammals, but their tissue distribution differs. HK1 is constitutively expressed in most normal tissues, HK2 is widely expressed in insulin-sensitive tissues, such as muscle and fat, and is also expressed in the kidney, blood vessels, and retina (72). Most immortalized and malignant cells show increased expression of HK2, which may contribute to an increase in glycolysis and provide energy and metabolites for tumor DNA synthesis (73). Existing research shows that the increase in HK2 activity is the initiator of glycolysis overload and downstream metabolic dysfunction, and the increase in HK2 and G-6-P causes metabolic dysfunction and accelerates cell aging and tissue damage (74).

Studies have shown that HIF-1α activation can upregulate the levels of HK1 and HK2 and that it plays an important role in tumor cell proliferation by enhancing glycolysis activity (75). Research shows that the increase in HK2 not only enhances glycolysis but also promotes EMT under hypoxia (76). Similar to GLUTs, HK2, a rate-limiting enzyme in glycolysis, is a potential target of glucose metabolism. Studies have proved that 3-Bromopyruvic acid and 2-deoxy-d-glucose can significantly inhibit HK activity and glycolysis (77).

### HIF Regulates PFK1

PFK1 converts fructose-6-phosphate (F-6-P) into fructose-1,6-diphosphate (FBP), which is the rate-limiting enzyme in glycolysis and the key control point for regulating glycolytic flux. PFK1 is a tetrameric protein with three subtypes: PFK-M (muscle), PFK-L (liver), and PFK-P (platelets). The dimerization of the PFK1 monomer is beneficial for maintaining the tertiary structure of the enzyme (78). The PFK1 dimer showed the lowest catalytic activity, while complete catalytic activity was achieved when it combined to form a tetramer (78). PFK1 is subject to complex allosteric regulation and is inhibited by high ATP concentrations and activated by adenosine monophosphate (AMP), which prevents excessive glucose degradation when ATP is sufficient. Moreover, it is inhibited by citrate and long-chain fatty acids produced in the TCA cycle (79). It must be said that the most effective allosteric activator of PFK1 is fructose- 2,6-diphosphate, and that the level of fructose- 2,6-diphosphate is closely related to that of fructose-6-phosphate-2-kinase/fructose-2,6-diphosphatase (PFK2, PFKFB) (79). PFKFB contains two catalytic domains with kinase and phosphatase activities and has dual functions. It increases the affinity of PFK1 to F-6-P, releases enzymes from ATP-mediated inhibition, and synergistically increases the affinity of PFK1 to AMP. Furthermore, PFKFB stabilizes PFK1 and promotes its association with tetramers and activates higher oligomers (80). Therefore, changes in the PFKFB activity are important for glucose metabolism.

PFKFB, an important regulator of glycolysis, has four isozymes (PFKFB1, PFKFB2, PFKFB3, and PFKFB4), among which PFKFB3 has approximately 700 times higher kinase activity than bisphosphatase activity, which is beneficial for the generation of intracellular F-2, 6-BP, and glycolysis (81). Studies have shown that HRE in the PFKFB3 promoter can be activated by HIF-1α under hypoxia, leading to an increase in glucose uptake and glycolysis flux. Glycolysis and pro-inflammatory activation of macrophages mainly depend on the accumulation of HIF-1α, which can increase glucose uptake and upregulate HK2 and PFKFB3 under hypoxia (82). In renal cell carcinoma, PFKFB3 knockout inhibits glycolysis and cell proliferation, suggesting that PFKFB3 could be used as a potential therapeutic target (83). Emerging evidence has shown that PFKFB3 also plays an important role in regulating physiological and pathological angiogenesis (84). Endothelial proliferation depends on glycolysis: 85% of ATP is produced by converting glucose into lactate, and pharmacological inhibition of PFKFB3 damages the development of the mouse retinal vascular system (85). In diabetic retinopathy, HIF-1α/PFKFB3 signaling not only promotes pathological angiogenesis, but also drives glycolysis, which damages the antioxidant capacity of neurons, leading to neuronal degeneration and an increase in the number of reactive glia (86). The kidney is a highly vascularized organ with a rich microvascular structure. Exploring the relationship between glucose metabolism in the renal microcirculation and renal fibrosis may provide new insights into understanding the mechanism underlying CKD development.

### HIF-1α Regulates PKM2

PK is the rate-limiting enzyme in glycolysis, which converts phosphoenolpyruvate (PEP) to pyruvate. PK has four subtypes (PKL, PKR, PKM1, and PKM2), which can be specifically expressed according to the different metabolic needs of tissues. PKR is only expressed in red blood cells, PKL is mainly expressed in the liver, PKM1 is mainly expressed in the skeletal muscle, heart, and brain, and PKM2, which exists in various differentiated tissues, is the main subtype in the kidney (87). According to reports, the expression level of PKM2 is increased in highly proliferating cells (such as stem cells and tumor cells), which is beneficial to the accumulation of glycolytic metabolites and stimulates tumor proliferation. PKM2 exists in four different enzyme states: inactive monomer, near-inactive dimer, inactive T-tetramer (low enzyme activity), and active R-tetramer (high enzyme activity). There is a conformational switch between the inactive T-state and active R-state during glycolysis (88).

PKM2 plays a key role in reprogramming tumor metabolism and is considered an important regulator of the Warburg effect. This downregulation reduces the glucose utilization and glycolysis rates and inhibits tumor growth (89). Research has shown that the change in the metabolic state of PKM2 is mainly mediated by the interaction between HIF-1α and c-Myc (an oncogenic transcription factor), which can upregulate the transcription of PKM2 and increase its protein synthesis (90). Wang et al. reported that HIF‐1α not only directly activates PKM2 but also upregulates the expression of PKM2 in an m5C-dependent manner by activating ALYREF (an m5C RNA binding protein), increasing glycolysis, and promoting bladder cancer cell proliferation (91). PKM2 is also involved in EMT, and the expression and nuclear accumulation of PKM2 increase when EMT is induced (92). Luo et al. demonstrated that PKM2 can also act as a co-activator, which can stimulate the transactivation of HIF-1α encoding GLUT1, LDHA, and PDK1 target genes in tumor cells by combining with the transactivation domain of HIF-1α, which accelerates glucose metabolic reprogramming and cancer progression mediated by HIF-1α (93).

Furthermore, increasing evidence indicates that PKM2 can be used as a biomarker of nephrotoxicity. In the conditioned medium of human TECs pretreated with cisplatin or other nephrotoxic substances (such as cyclosporine A and HgCl2), the secretion of PKM2 increased, and this effect was not observed in liver and breast cancer cells, suggesting that PKM2 is specific to renal cells and can be used as an early marker of nephrotoxicity (94). It is well established that hyperglycemia promotes CKD. Hyperglycemia reduces the formation and activity of the PKM2 tetramer in the glomerulus, TECs, and podocytes through sulfinylation, resulting in the increase of toxic glucose metabolites and mitochondrial dysfunction (95, 96). The activation of PKM2 can restore mitochondrial function by inhibiting the accumulation of toxic glucose metabolites, increasing glycolysis and TCA cycle flux, and inducing mitochondrial biogenesis (96). However, the protective role of glycolysis flux may be relative, and extreme or long-term hyperglycemia may alter this protective mechanism. In diabetic nephropathy, knocking out sirtuin 3 can induce TGF-β/smad3 signaling, which increases the accumulation of HIF-1α and formation of PKM2 dimers, and enhances the transcription of glycolytic enzymes, leading to the accumulation of glycolytic intermediates before pyruvate, induces abnormal glycolysis, and helps to drive EMT, thus accelerating renal fibrosis (97). Hence, targeting PKM2 may be a new strategy to prevent or treat kidney diseases.

## HIF-1α Inhibits OXPHOS

HIF-1α upregulates glycolytic enzymes and reduces the amount of metabolites entering the TCA cycle by upregulating pyruvate dehydrogenase kinases (PDKs), thus inhibiting OXPHOS (98). PDKs can inhibit the activity of PDC, which is an important nodal enzyme that catalyzes the conversion of pyruvate into acetyl-CoA and is connected to the TCA cycle (98). There are four subtypes of PDKs (PDK1, PDK2, PDK3, and PDK4) with different distributions and binding affinities. PDK2 and PDK4 are the most widely distributed in the heart, liver, and kidney, while the levels of PDK1 and PDK3 are limited in tissues (99). Regarding binding affinity, PDK4 has the lowest affinity, PDK3 has the highest affinity, PDK1 and PDK2 have medium affinity, and PDK1-3 can interact with various signal transduction factors, which is helpful in cancer progression (99). However, PDK4 can act as both an oncogene and tumor suppressor (99).

Research shows that HIF-1α can effectively inhibit PDC activity by activating the transcription of PDK1, preventing pyruvate from being converted into acetyl-CoA, thus hindering the start of the TCA cycle (100). Notably, the blocking effect of HIF-1α on the TCA cycle not only activates PDK1 but also promotes LDHA expression (101). LDHA is the key enzyme responsible for converting pyruvate into lactate in the last step of glycolysis, and it also produces NADH2, which is a cofactor necessary for maintaining glycolytic activity (102). HIF-1α can increase lactate by activating LDHA, reducing the conversion of pyruvate into acetyl-CoA, and further keeping pyruvate away from the TCA cycle (101). He et al. selected six active molecules from a database of 500,000 molecules using high-throughput virtual screening, optimized their structures, and developed a new dual-target molecule with nanomolar activity against PDK1 and LDHA. The results showed that this molecule inhibited the activity of PDK1 and LDHA, significantly reduced the production of lactate in tumor cells, increased oxygen consumption, and regulated glucose metabolism (103).

Moreover, HIF-1α interacts with other transcription factors to regulate glucose metabolism. c-Myc regulates cell metabolism and cell proliferation, either alone (under normal oxygen) or in combination with HIF-1α, and can activate PDK1 and LDHA, increase the conversion of pyruvate to lactate, and weaken mitochondrial respiration (104). Estrogen-related receptors (ERRs) are orphan nuclear receptors that regulate cell energy metabolism by coordinating mitochondrial biogenesis and OXPHOS (105). Cai et al. showed that ERRs can be combined with the promoter of glycolytic genes alone to activate gene transcription, and can also be combined with c-Myc to activate the transcription of glycolytic genes. At the same time, ERRs can also be used as a cofactor of HIF-1α to enhance HIF-1α-dependent transcription of glycolytic genes under hypoxia, thus contributing to the Warburg effect (106).

Generally, HIF-1α-dependent transcription reduces PDH activity by upregulating PDK1 and increases the LDH-mediated conversion of pyruvate to lactate, thus reducing pyruvate to the TCA cycle effectively, leading to a decrease in TCA cycle flux and OXPHOS. In view of this, PDK1 can be used as an effective target for precise therapy, in which the pyruvate analog dichloroacetate, a naturally occurring inhibitor, has been shown to inhibit PDK activity *in vitro* and *in vivo (* 107). Unfortunately, there are no reports on the effect of HIF-1α on OXPHOS in renal fibrosis. In the future, more in-depth research in this area should be conducted.

## HIF-1α mediates phosphoinositide 3-Kinase (PI3K)/AKT glycolysis regulation

PI3K is an intracellular heterodimer lipid kinase that responds to extracellular stimuli, such as growth factors, cytokines, and hormones, and participates in regulating cell survival, proliferation, and metabolism (108). Akt, a serine/threonine kinase, is the central molecule of the PI3K pathway, which can be combined with lipid products of PI3K and thus localize on the cell membrane to activate many cell processes, such as glucose metabolism, apoptosis, and cell proliferation (109). Many studies have shown that PI3K/Akt directly or indirectly upregulates glycolytic enzymes through post-translational modifications (such as phosphorylation). For example, Akt phosphorylation can regulate GLUT1 expression and increase glucose uptake (110). Akt also enhances the phosphorylation level of glycolytic enzymes, such as HK2 and PFK1, and increases the glycolysis rate (110). Moreover, Akt indirectly coordinates glycolysis and the TCA cycle by inducing the expression of HIF-1α (111). The transcription factor forkhead box O3a directly inhibits c-Myc transcription. Akt indirectly activates c-Myc by phosphorylating forkhead box O3a, upregulates glycolytic enzymes induced by c-Myc, and catalyzes glycolysis (112). Therefore, the activation of PI3K/Akt is closely related to the glycolytic activity of cells.

**Figure 3:**
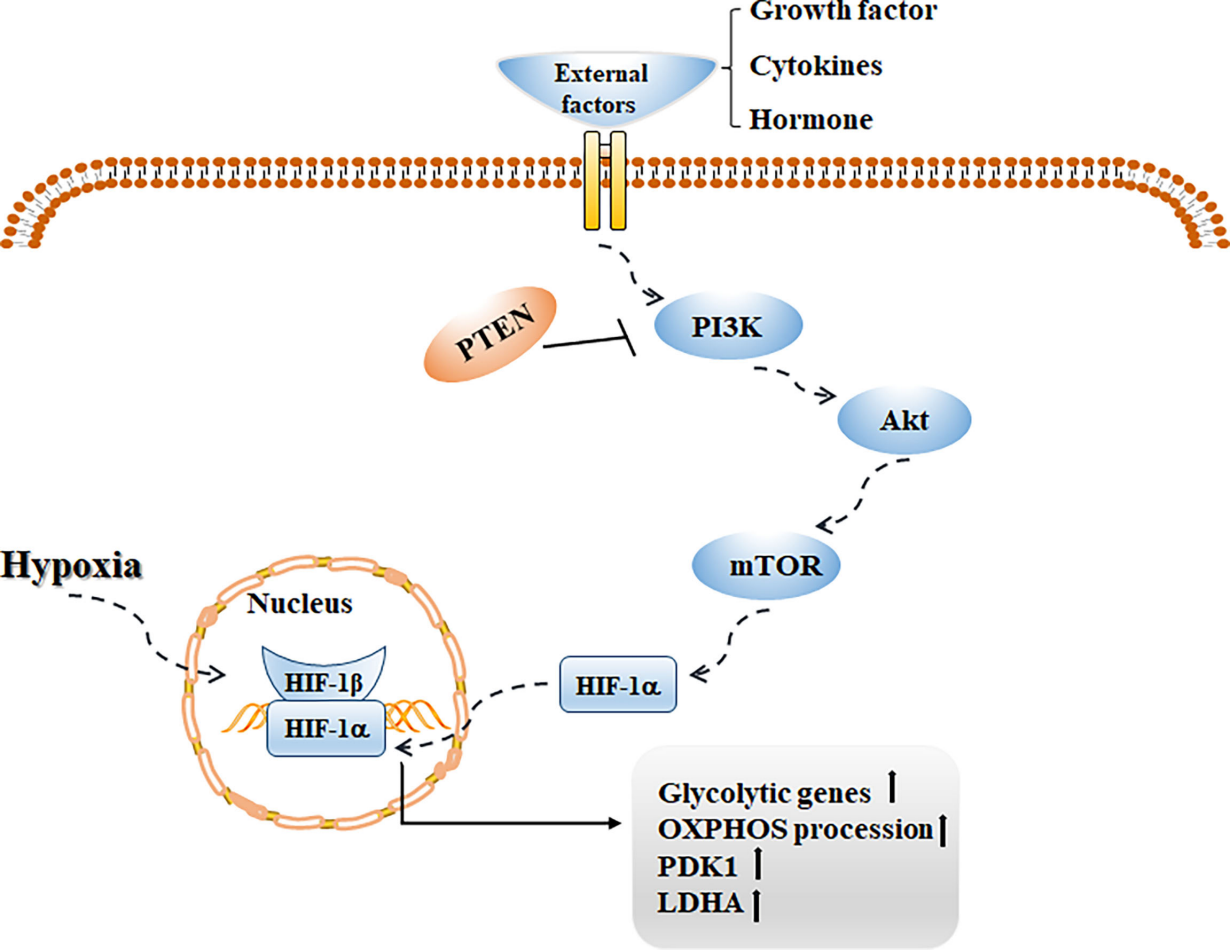
With the stimulation of external factors such as growth factor, cytokines and hormone, PI3K/Akt/mTOR signaling pathway is activated, which can promote HIF-1α transcription activity, increase the transcription of glycolytic genes, up-regulate the levels of PDK1 and LDHA, and then increase glycolysis while inhibit OXPHOS.

mTOR is a conserved serine/threonine kinase that regulates anabolism and cell growth, and its phosphorylation level can be regulated by Akt (113). Studies have shown that mTOR mediated by the PI3K/Akt pathway is of great significance in regulating cell survival, metabolism, and angiogenesis. Abnormalities in this pathway are common in human tumor diseases, and targeting cytokines in this pathway is helpful in increasing the therapeutic effect on tumors (114). Notably, this pathway plays a key role in promoting glycolysis and lactate production. Activating this signaling pathway can increase the transcription of glycolytic genes by promoting HIF-1α-dependent mechanisms, upregulating the activities of PDK1 and LDHA, and inhibit OXPHOS (115) (**Figure 3**). Weng et al. first confirmed that fasting upregulated the level of the cholesterol steroid gene farnesyl-diphosphate farnesyl transferase 1 (FDFT1) and then decreased glycolysis by negatively regulating AKT/mTOR/HIF1α signaling, thus prolonging the survival time of colorectal cancer patients (116). Phosphatase and tensin homolog deleted on chromosome ten (PTEN) is a tumor suppressor, and its biological effect is mainly the negative regulation of PI3K signaling (117). Other studies have shown that deletion of PTEN leads to many metabolic changes (118). Xiao et al. confirmed that overexpression of miR-21 can mediate the PI3K/Akt/mTOR axis by increasing PTEN and then reducing the expression of HIF-1α, resulting in a decrease in aerobic glycolysis in bladder cancer cells (119). Interestingly, some studies have shown that the metabolic pathway mediated by PI3K signaling also plays an important role in tissue fibrosis. Hu et al. reported that lipopolysaccharide upregulated the expression of PFKFB3 by activating the PI3K/Akt/mTOR pathway, increasing aerobic glycolysis in lung fibroblasts, promoting collagen synthesis, and accelerating pulmonary fibrosis (120). Studies have shown that activating the PI3K/Akt/mTOR and HIF-1α signaling pathways can effectively inhibit inflammatory reactions, reduce ECM deposition, and reverse EMT, and thus play a protective role in renal fibrosis (121). The possible pathogenic mechanism underlying the glycolysis process of renal fibrosis needs to be elucidated.

## Effects of HIF stabilizers and inhibitors on the kidney

HIFs, a key factor in the metabolic regulation of eukaryotic cells under hypoxia, controls the expression of downstream target genes, involving multiple links such as oxygen transport, angiogenesis and cell metabolism, and plays an important regulatory role in diseases. At present, the clinical application of HIFs mainly focuses on two aspects: the stabilizers of HIF to treat renal anemia, and HIF inhibitors for the treatment of tumors.

CKD often progresses to end-stage renal disease, and most patients suffer renal anemia. The pathogenesis of anemia is multifactorial, including chronic inflammation, iron deficiency, and shortened half-life of erythrocytes, and the deficiency of erythropoietin is the primary cause (122). Erythropoiesis-stimulating agents combined with oral or intravenous iron supplementation is the standard treatment for renal anemia, however, some safety reports have demonstrated that high-dose erythropoiesis-stimulating agent therapy is associated with increased risk of cardiovascular events (123). Roxadustat (FG-4592) is an oral HIF prolyl hydroxylase inhibitor (HIF-PHI) that stimulates erythropoiesis and regulates iron metabolism. It has been approved in China for the treatment of dialysis-dependent (124) and non-dialysis-dependent CKD (125). Although clinical data have demonstrated that Roxadustat is effective and well-tolerated, information is still required on the range of non-erythropoiesis effects of HIF stabilizers, such as a potential proangiogenic effect related to the VEGF and VEGF receptors, the progression of pulmonary arterial hypertension (55), and the potential development of renal fibrosis related to the HIF activation over long periods (126). Considering that HIFs regulate a broad spectrum of genes, HIF-PHI are expected to protect against metabolic disorders in addition to erythropoiesis. Sugahara et al. determined the potential renoprotective effects of a HIF-PHI, enarodustat, which can improve glucose and lipid metabolism in obese and diabetic mice and reduce the infiltration of macrophages in kidney and adipose tissue (127). Studies have shown that the metabolites of glycolysis and the TCA cycle were accumulated and amino acids were reduced in kidney tissues of diabetic animals, and these metabolic disturbances were alleviated by enarodustat. Meanwhile, enarodustat can also reduce oxidative stress by increasing the ratio of glutathione to glutathione disulfide (128). Thus, stabilizing HIF can counteract the changes of renal energy metabolism in early diabetic nephropathy.

HIF has recently been recognized as an important drug target for cancer treatment. Many studies have determined that the increase of HIF level promotes the formation of tumor microvessels, tumor cell proliferation, cell survival and progression, and metastatic spread (129). There are two major categories of HIF inhibitors: small-molecule inhibitors that directly affect the expression or function of HIF, and indirect HIF inhibitors that block other molecules in related signaling pathways. Clear cell renal cell carcinoma (ccRCC) is the most common subtype of renal cell carcinoma, and the inactivation of VHL gene is the main molecular feature. The loss of VHL leads to the accumulation of HIF-α and then the uncontrolled activation of HIF target genes, many of which promote angiogenesis, cell proliferation and metastasis of ccRCC (130). A large number of clinical trials have indicated that drugs that inhibit HIF/VEGF signaling demonstrated overall survival advantages for patients with advanced or metastatic renal cell carcinoma (131).The latest research focuses on Belzutifan, an inhibitor of HIF-2α, which was first approved in the USA to treat solid tumors including ccRCC and von Hippel-Lindau disease-associated renal cell carcinoma (132).

## Conclusions

In recent years, the annual incidence of CKD has been on the rise worldwide. Renal fibrosis is a dynamic process driven by many complicated factors and a key pathogenic factor that leads to end-stage renal disease. However, there is no clinical treatment or drug available to cure or reverse renal fibrosis. Exploring the pathogenesis of renal fibrosis and blocking or reversing the renal fibrosis process is of great clinical significance. Changes in energy metabolism in various diseases have become a hot research topic. Recent studies have shown that there is a metabolic pattern similar to that of tumor cells in the process of renal fibrosis, that is, a switch from OXPHOS to aerobic glycolysis. After kidney injury, enhancing aerobic glycolysis is a compensatory energy supply mechanism. Therefore, it is of great significance to study the related proteins that change during glucose metabolism. HIF-1α is a key transcription regulatory factor that mediates the adaptive response of cells to hypoxic microenvironments. It promotes renal fibrosis by regulating inflammatory reactions, promoting EMT, activating the transcription of downstream cytokines related to fibrosis, and interacting with fibrogenic signaling pathways. It is worth noting that HIF-1α-dependent glucose metabolism reprogramming plays an important role in the adaptive response of cells, which is mainly manifested by upregulating the expression of glucose transporters and glycolysis-related enzymes, promoting glycolysis while inhibiting OXPHOS. In summary, targeting metabolic pathways, especially HIF-1α-mediated aerobic glycolysis, may be beneficial in renal fibrosis. Second, an in-depth study of glycolysis inhibitors will be a reliable means of improving renal fibrosis. However, it should be noted that the specific influence of anti-fibrotic treatments on renal function and their final curative effects, the best action time and dose, and other issues need to be clarified, for patients to obtain the greatest benefits.

## Author contributions

YD and XW conceived and designed the review; XW wrote the original draft; YH updated the references; ML and LJ wrote the conclusions and sections of the manuscript; All authors have read and agreed to the published version of the manuscript.

## Funding

This research was funded by Finance Department of Jilin Province, grant number JLSWSRCZX2021-006.

## Conflict of interest

The authors declare that the research was conducted in the absence of any commercial or financial relationships that could be construed as a potential conflict of interest.

## Publisher’s note

All claims expressed in this article are solely those of the authors and do not necessarily represent those of their affiliated organizations, or those of the publisher, the editors and the reviewers. Any product that may be evaluated in this article, or claim that may be made by its manufacturer, is not guaranteed or endorsed by the publisher.
